# A head-to-head comparison of well-being of older people (WOOP) and EQ-5D-5L in patients, carers and general public in China

**DOI:** 10.1038/s41598-023-33248-2

**Published:** 2023-04-17

**Authors:** Xun Ran, Zhuxin Mao, Zhihao Yang

**Affiliations:** 1 Department of Clinical Medicine, Guizhou Medical University, Guiyang, China; 2 Centre for Health Economics Research and Modelling Infectious Diseases, Vaccine and Infectious Disease Institute, University of Antwerp, Antwerpe, Belgium; 3 Department of Health Services Management, Guizhou Medical University, Guiyang, China; 4 Center of Medicine Economics and Management Research, Guizhou Medical University, Guiyang, China

**Keywords:** Health care economics, Quality of life

## Abstract

Recently, well-being of older people measure (WOOP) was developed and validated in a Dutch population. Although WOOP was developed targeting the older people, it has the potential for use in a wider population. In this study, we aimed to examine the relationship between WOOP and EQ-5D-5L and compared their psychometric properties in a sample of patients, carers and healthy general public covering a wider age group. We conducted a cross-sectional study in Guizhou Province, China between July and August 2022. Data was collected using paper and pencil. We analysed and reported the acceptability, item response distribution, the Spearman correlation coefficients of all items, the Exploratory Factor Analysis (EFA) of all items, the known-group validity and the convergent validity of EQ-5D-5L utility and WOOP utility. A total of 322 participants completed the survey with 105 patients, 101 carers and 116 healthy general public. 9% of participants had at least one missing response. Three items of WOOP did not have any level 5 responses and EQ-5D-5L had more level 1 responses. The correlations were low between EQ-5D-5L and WOOP items and the three-factor EFA showed these two instruments had only one shared factor and the other two factors were only related to WOOP items. Younger people had lower missing response rate and a different response distribution for three items. WOOP measures a broader construct beyond health while EQ-5D-5L is a more sensitive instrument when health is considered alone. There is a potential of using WOOP in a wider population.

## Introduction

The finite resources have led to an increasing need for economic evaluation within health and social care decision-making to optimize resource allocation strategies^1^. Economic evaluation considers both costs and consequences of alternative courses of action, while Quality-adjusted life-year (QALY) has become a widely used outcome measure^1^. Preference-based multi-attribute utility instruments (MAUIs), most of which were developed aiming to describe and measure the construct of health, are routinely used to estimate QALY^2,3^. For example, EQ-5D, which is the most commonly used MAUI^4^, includes five health-related dimensions (mobility, self-care, usual activities, pain/discomfort, and anxiety/depression) to describe health status^5^. EQ-5D, which includes both EQ-5D-3L and EQ-5D-5L, is commonly used in China to assess population health^6,7^ and quantify disease burden^8,9^. With two additional response levels, EQ-5D-5L has demonstrated superior measurement properties compared to EQ-5D-3L worldwide^9–11^. Moreover, EQ-5D is the recommended instrument for calculating health utility^4^, and the value sets for both versions of EQ-5D have been established in China ^12,13^.

It can be argued that the ultimate goal of health and social care is to improve health, however, as a recent qualitative review reported, some aspects of quality of life (QoL) can go beyond health and are relevant to patients, informal caregivers, and social care users^14^. These aspects may be equally important when considering evaluating health and social care interventions^15^. Consequently, to capture other relevant and important aspects of quality of life (QoL) other than health, various well-being measures have been developed for use in assessing broader benefits of health and social care.

Well-designed well-being measures are useful to rationalize resource allocation in health and social care in China, however, limited well-being measures are available for use in Chinese populations. The ICECAP-A (ICEpop CAPability measure for adults) was adapted in Chinese^16^, but was found to perform less satisfactorily compared to EQ-5D and AQoL-7D^17,18^ and has not been frequently used in practice in China. The Short Warwick–Edinburgh Mental Well-Being Scale (SWEMWS) has been translated into Chinese and has been demonstrated in the general population and hospitalized patients^19,20^, but it focuses more on positive mental well-being and does not aim to estimate utilities. EuroQol Health and Well-being (EQ-HWB) has been developed internationally and included Chinese participants in its development process, but the conceptual framework needs further investigation in China^21^. Following an in-depth investigation of older populations’ understandings of well-being, well-being of older people measure (WOOP) was recently developed and validated in a Dutch population^22^. The measure incorporates nine items (physical health, mental health, social life, receiving support, acceptance and resilience, feeling useful, independence, making ends meet and living situation), and each of them was considered important when constituting well-being^22^. It has now been translated, adapted and validated for use in a Chinese older adult population. The content of the measure is comprehensive, yet not too lengthy, thus well-suited for self-completion.

Although WOOP was developed targeting the older adult population, it has the potential for use to assess well-being in a wider population. This is because, first, the included items in WOOP are not population-specific and most of the items were found to be important in patients, informal caregivers, and general populations^14,23^. Empirical studies have also found comparable measurement properties between the well-being measures (ICECAP family) developed for adults and for older people^24^. Strong correlations between the two measures at item level were also reported, indicating similar concepts behind the questionnaire items^24^. In fact, the generic well-being measure ICECAP-A, was developed based on the older people version, ICECAP-O (ICECAP for older people) and the included items are largely overlapping between the two measures^25,26^.

In this study, we aimed to investigate whether WOOP is appropriate for use as a more generic well-being measure for use in patients, carers and general public in China by evaluating how WOOP is related to the commonly used EQ-5D-5L and comparing the psychometric properties of these two instruments. This paper also aims to explore the use of WOOP in non-elderly populations.

## Method

### Sampling

The data collection was carried out in Guizhou Province, China between July and August 2022. For patients and carers, we recruited those who were using inpatient services in the Affiliated Hospital of Guizhou Medical University. A convenient sample of patients and carers were mainly recruited from the Hepatobiliary Surgery, Thoracic Surgery and Anorectal Surgery departments, covering both pre-surgery and post-surgery patients. Carers who were attending patients in these three departments were invited to participate. For general public, a representative sample of Chinese general population was recruited using quota sampling method. Quotas used were gender, age, education and Hukou (rural vs urban). For all participants, other inclusion criteria were (1) either being able to read the questionnaire or able to converse in the local language; (2) gave informed consent. This study was approved by the Ethics Committee of Guizhou Medical University (Approval letter No. 276-2022). The study was conducted in accordance with the principles of Declaration of Helsinki and approved guidelines.

### Instruments

WOOP has nine items: physical problem, mental problem, social contacts, receive support, acceptance and resilience, feeling useful, independence, making ends meet and living situation, each with five response levels corresponding to excellent, good, fair, poor and bad performances^27^. WOOP responses can also be converted into utility on a QALY scale and so far, only one value set was established^28^. EQ-5D-5L is composed of two parts: a five-item health descriptive system and the EQ visual analogue scale (EQ-VAS)^29^. The EQ-5D-5L descriptive system comprises five dimensions: mobility, self-care, usual activities, pain/discomfort, and anxiety/depression, and five response levels: no problems, slight problems, moderate problems, severe problems and extreme problems/unable to for each dimension^30^. In 2017, the value set for EQ-5D-5L was established using the preferences of 1271 urban residents from five cities. The value set comprises the utility value of 3125 EQ-5D-5L health states, ranging from −0.391 (for health state 55,555) to 0.955 (for the second-best state 11,211). Details of the value set development process have been described elsewhere^12^. EQ-VAS, as part of the EQ-5D-5L instrument, assesses the overall health and has demonstrated satisfactory performance^31^. Notably, both instruments use a recall period of today, which avoids the bias introduced by different recall periods.

### Data collection procedure

The survey was conducted using paper and pencil. Three undergraduate students were recruited from Guizhou Medical University. All interviewers received standardized data collection training on the background of this study, and the introduction of the instruments. A written informed consent was obtained before responding to the questionnaire. Respondents were encouraged to respond to the questionnaire by themselves, interviewers were able to explain the questions if necessary. Notably, respondents were instructed not to respond if they could not understand the item or felt the items were irrelevant.

### Data analysis

To understand the relationship between EQ-5D-5L and WOOP and compare their psychometric properties, we analysed and reported the acceptability, item response distribution, the Spearman correlation coefficients of all items, the Exploratory Factor Analysis (EFA) of all items, the known-group validity and the convergent validity of EQ-5D-5L utility and WOOP utility.

The acceptability of these two instruments was measured by the number of participants who requested explanations during the data collection process and the number of missing responses. Next, we reported the response distribution for each item. Specifically, items with over 70% of responses using level 1 (excellent) and level 5 (bad) suggesting a high ceiling effect and floor effect respectively^21^. To understand the relationship between the WOOP and EQ-5D-5L, pairwise spearman correlations were reported for all items. Correlation strength was defined as: trivial: < 0.10; small: 0.10–0.29; moderate: 0.30–0.49; high: 0.50–0.69; very high: 0.70–0.89; perfect: > 0.90^22^. High and perfect correlations suggest items may be measuring the same construct and an overall low correlation suggests the discriminant validity of these two instruments^21^. Since WOOP has one item to measure mental health and physical health respectively, we hypothesized that the first four items of EQ-5D-5L would have a high correlation with the physical health item of WOOP, while the anxiety/depression item of EQ-5D-5L would have a high correlation with the mental health item of WOOP items. Exploratory factor analysis (EFA) was used to further understand the relationship between items. Both Bartlett's test for sphericity and Kaiser–Meyer–Olkin (KMO) measure of sampling adequacy were estimated prior to EFA. A parallel analysis was used to determine the number of factors. Only the highest factor loading was reported for each item, which needed to be ≥|0.40|^22^.

For known-group validity and convergent validity, we first calculated the utility of both instruments and plotted their distributions. EQ-5D-5L utility was calculated based on the value set of China^12^ and WOOP utility was calculated based on the Dutch value set, which is the only available value set^28^. Four known-group were defined as: patients vs general public, patients vs carers, EQ-VAS < 80 vs EQ-VAS ≥ 80, and age < 60 vs age  ≥ 60. We hypothesized that patients would report worse outcomes when compared with general public and carers, and individuals who reported lower EQ-VAS and who were older would report worse outcomes. For reference, we also examined the difference between general public vs carers and hypothesized a null effect. Cohens D effect size was used to quantify the known-group validity following the criteria: 0.2 to 0.5 suggests small, 0.5 to 0.8 suggests medium, and  ≥ 0.8 or more suggests large effect sizes, respectively. Convergent validity was examined by Pearson correlation between the utilities and EQ-VAS^22^.

WOOP was developed to measure the well-being of older people, yet the item descriptions are not specific to the older population. To explore the potential of using WOOP in the younger population, we examined the proportion of missing responses by age group. Our hypothesis is that WOOP can be used for the younger population and the younger population would have fewer problems using this instrument, that is, those aged ≤ 60 years would be less likely to have missing responses compared to the older group (aged > 60 years). We also plotted the item distributions of WOOP items for those who were above 60 years and for those who were below, we tested the difference using Chi^2^ test.

## Results

In total, 322 participants participated the study and completed the survey, with 105 patients, 101 carers and 116 healthy general public. The mean age of our sample was 47.89 (SD: 17.87) years old and 55.59% of which being male, 72.36% of which being the Han ethnic group. Education level was evenly distributed across four categories. Table 1 shows detailed demographic information our sample and reports the WOOP utility, EQ-5D utility and EQ-VAS score for the whole sample and by subgroup. The mean utilities measured by WOOP and by EQ-5D (0.874 vs 0.880) were similar for the whole sample but differed in subgroups, with EQ-5D having higher utilities for carers (0.906 vs 0.947) and general public (0.887 vs 0.947) but had lower utility for patients (0.823 vs 0.718) when compared with WOOP. Both WOOP utility and EQ-VAS suggested carers reported better outcomes.

**Table 1:** Demographic information of the sample, n = 322.

	Whole sample, N = 322		Patients, n = 105		Carers, n = 101		General public, n = 116	
	n	%	n	%	n	%	n	%
Gender								
Male	179	55.59	67	63.81	52	51.49	60	51.72
Female	143	44.41	38	36.19	49	48.51	56	48.28
Age group								
< 30	67	20.81	6	5.71	33	32.67	28	24.17
30–59	163	50.62	43	40.95	51	50.5	69	59.48
≥ 60	92	28.57	56	53.33	17	16.83	19	16.38
Ethnicity								
Han	233	72.36	91	86.67	75	74.26	67	57.76
Minority	89	27.64	14	13.33	26	25.74	49	42.24
Education*								
Primary or lower	77	24.06	31	29.81	13	13.00	33	28.45
Junior high or equivalent	93	29.06	32	30.77	17	17.00	44	37.93
Senior high or equivalent	83	25.94	23	22.12	36	36.00	24	20.69
University or higher	67	20.94	18	17.31	34	34.00	15	12.93
WOOP utility: Mean, SD	0.874, 0.147		0.823, 0.207		0.906, 0.107		0.887, 0.106	
EQ-5D utility: Mean, SD	0.880, 0.318		0.718, 0.318		0.947, 0.107		0.947, 0.088	
EQ-VAS: Mean, SD	83.00, 13.42		77.73, 16.57		85.99, 11.89		84.53, 10.62	

*With one missing response from the patient group and carer group respectively.

^#^WOOP utility, EQ-5D utility and EQ-VAS were based on the sample without any missing responses, n = 293.

Twenty-nine participants (9.01% of the whole sample) had at least one missing response. Specifically, ‘acceptance and resilience’ had 5, ‘feeling useful’ had 4, both ‘social contacts’ and ‘making ends meet’ had 3, ‘receive support’ had 2 and ‘independence’ had 1 missing responses, respectively for WOOP. Only ‘self-care’ had 2 missing responses for EQ-5D.

**Figure 1:**
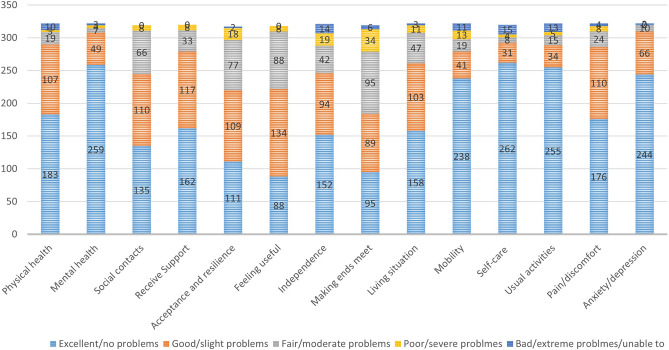
Response distributions of EQ-5D-5L and WOOP.

Figure 1 shows the response distributions WOOP and EQ-5D for the whole sample. All five dimensions of EQ-5D showed higher proportions of level 1 responses, with pain/discomfort having the least proportion of level 1 responses (54.66%) and self-care having the highest proportion of level 1 responses (81.88%). For WOOP, both the physical health and mental health had a high proportion of level 1 responses, especially for mental health, the percentage of level 1 responses accounted for 80.43% of the whole responses. For other WOOP items, level 1 to 3 responses accounted for the majority of the responses. ‘Social contact’, ‘receive support’ and ‘feeling useful’ did not have any level 5 response.

Table 2 shows the Spearman correlations between WOOP and EQ-5D items. Overall, the correlations between EQ-5D and WOOP were mostly small. Very high correlations were presented within EQ-5D items, i.e., ‘usual activities’ correlated with ‘mobility’ (0.735) and ‘self-care’ (0.802) respectively. In addition, ‘mobility’ presented a high relationship between ‘pain/discomfort’ and ‘self-care’ respectively. The only high correlation for WOOP was reported between ‘social contact’ and ‘receive support’ (0.590). ‘Receive support’ and ‘making ends meet’ showed the most trivial correlation with other items.

**Table 2:** Spearman correlations between WOOP and EQ-5D-5L items, n = 322.

	WOOP									EQ-5D-5L			
	Physical health	Mental health	Social contacts	Receive Support	Acceptance and resilience	Feeling useful	Independence	Making ends meet	Living situation	Mobility	Self-care	Usual activities	Pain/discomfort
Physical health													
Mental health	0.350												
Social contacts	0.195	0.389											
Receive Support	0.131	0.282	**0.590**										
Acceptance and resilience	0.370	0.293	0.353	0.250									
Feeling useful	0.260	0.235	0.406	0.366	0.444								
Independence	0.292	0.265	0.270	0.260	0.484	0.413							
Making ends meet	*0.078*	*0.098*	0.282	0.206	0.248	0.293	0.251						
Living situation	0.172	0.249	0.371	0.350	0.287	0.261	0.187	0.409					
Mobility	0.378	0.213	0.111	* − 0.020*	0.255	0.217	0.253	*0.061*	0.147				
Self-care	0.344	0.246	0.144	*0.036*	0.260	0.221	0.381	*0.058*	*0.070*	**0.690**			
Usual activities	0.345	0.260	0.172	*0.043*	0.266	0.246	0.334	*0.071*	0.128	**0.735**	**0.802**		
Pain/discomfort	0.483	0.247	0.215	*0.082*	0.300	0.207	*0.099*	0.156	0.203	**0.509**	0.413	0.428	
Anxiety/depression	0.312	0.442	0.370	0.181	0.422	0.318	0.304	0.237	0.262	0.205	0.236	0.224	0.321

*Bold suggests high correlation, italics suggests trivial correlation.

Factor tests reported a KMO of 0.852 and a *P* value = 0.001 for the Bartlett's sphericity test, suggesting our sample is suitable for factor analysis. The parallel analysis suggested a three-factor model, which was shown in Table 3. The first factor included ‘physical health’ and ‘mental health’ from WOOP, ‘mobility’, ‘usual activities’ and ‘pain/discomfort’ and ‘anxiety/depression’ from EQ-5D; the second factor included ‘social contact’ and ‘receive support’ from WOOP; the third factor included ‘independence’ and ‘making ends meet’ from WOOP. Both the second and third factors were measured by WOOP only. These three factors explained 49.6%, 30.6% and 19.4% of variance respectively. ‘Acceptance and resilience’, ‘living situation’ and ‘feeling useful’ all had high uniqueness values and did not load on any factors.

**Table 3:** Three-factor EFA results.

Item	Factor 1	Factor 2	Factor 3	Uniqueness
**WOOP**				
Physical health	0.725			0.465
Mental health	0.630			0.589
Social contacts		0.841		0.276
Receive Support		0.899		0.163
Acceptance and resilience				0.754
Feeling useful				0.854
Independence			0.679	0.632
Making ends meet			0.761	0.357
Living situation				0.897
**EQ-5D-5L**				
Mobility	0.834			0.302
Self-care				0.998
Usual activities	0.829			0.302
Pain/discomfort	0.603			0.621
Anxiety/depression	0.485			0.729
**Total variance explained**	0.496	0.306	0.194	
**Correlation with Factor 2**	0.344			
**Correlation with Factor 3**	0.188	0.334		

**Figure 2:**
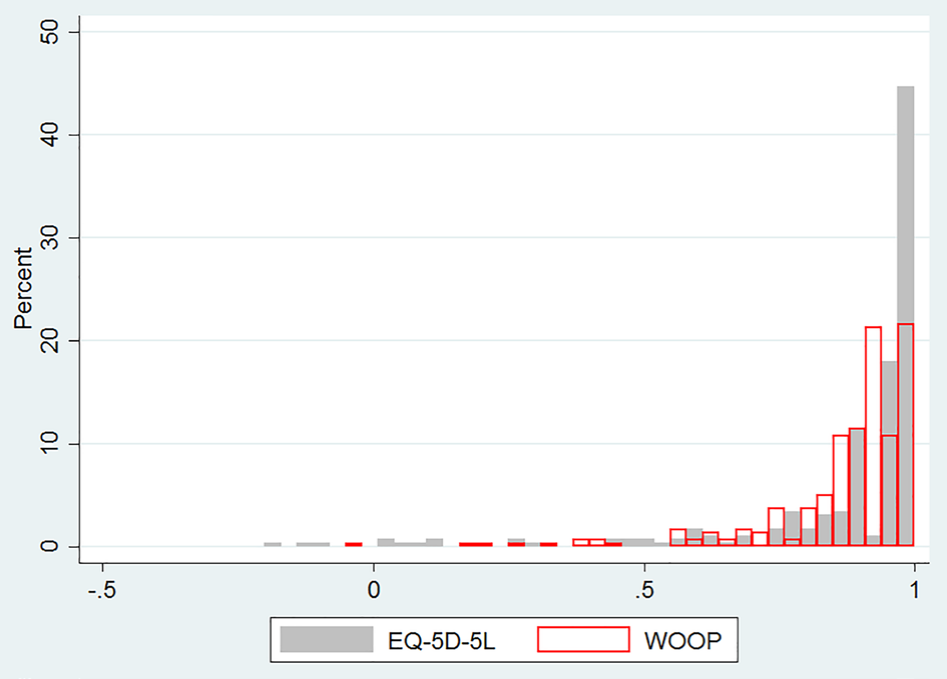
Utility distributions of EQ-5D-5L and WOOP.

The utilities of both instruments are left skewed, with EQ-5D-5L has a clustering at 1. Figure 2 shows the utility distributions of these two instruments and Table 4 shows the known-group validity of the WOOP and EQ-5D-5L utilities. The effect sizes of EQ-5D utility were higher than the WOOP utility except for the group of carer vs healthy, for which, both instruments did not show meaningful effects. Overall, EQ-5D utilities showed large variations, with the patient group having the lowest mean utility of 0.718 and the healthy group having the highest mean utility of 0.947. In comparison, the variations of WOOP utilities were smaller, ranging from 0.823 (for patients) to 0.906 (for carers). The Pearson correlation coefficients were 0.446 and 0.403 between WOOP utility, EQ-5D utility and EQ-VAS respectively. The Pearson correlation coefficient between WOOP utility and EQ-5D utility was 0.643.

**Table 4:** Known-group validity results.

	n	EQ-5D-5L utility	WOOP utility
**Patient vs Carer**			
Patient	86	0.718, 0.318	0.823, 0.207
Carer	94	0.947, 0.107	0.906, 0.107
Effect size		−0.981	−0.513
**Patient vs healthy**			
Patient	86	0.718, 0.318	0.823, 0.207
Healthy	113	0.947, 0.088	0.887, 0.106
Effect size		−1.045	−0.405
**Carer vs Healthy**			
Carer	94	0.947, 0.107	0.906, 0.107
Healthy	113	0.947, 0.088	0.887, 0.106
Effect size		−0.05	0.182
**EQ-VAS**			
≥ 80	162	0.926, 0.163	0.907, 0.094
< 80	131	0.822, 0.258	0.834, 0.186
Effect size		0.494	0.511
**Age group**			
≥ 60	74	0.780, 0.318	0.834, 0.205
< 60	219	0.914, 0.157	0.888, 0.118
Effect size		−0.639	−0.372

**Figure 3:**
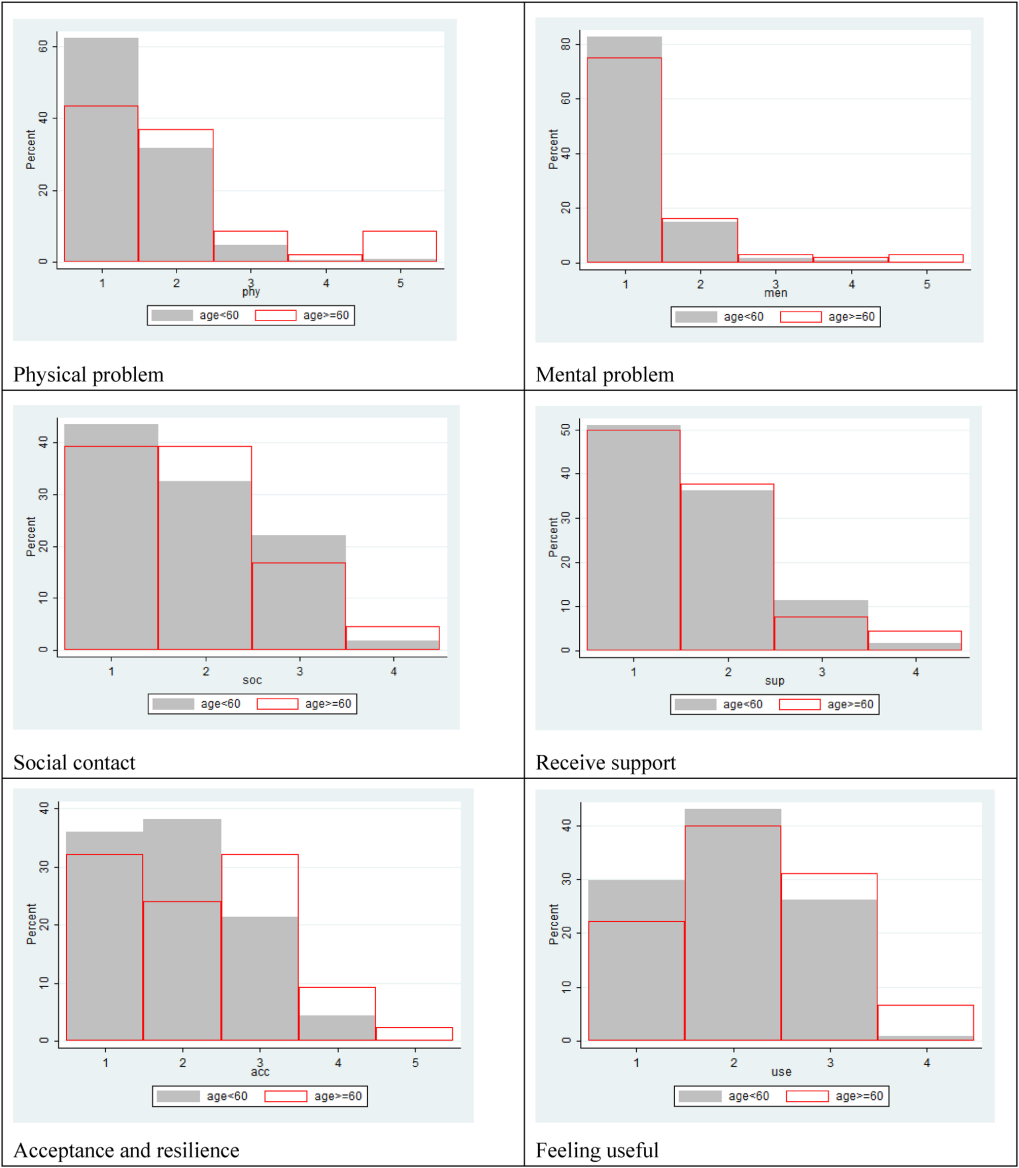

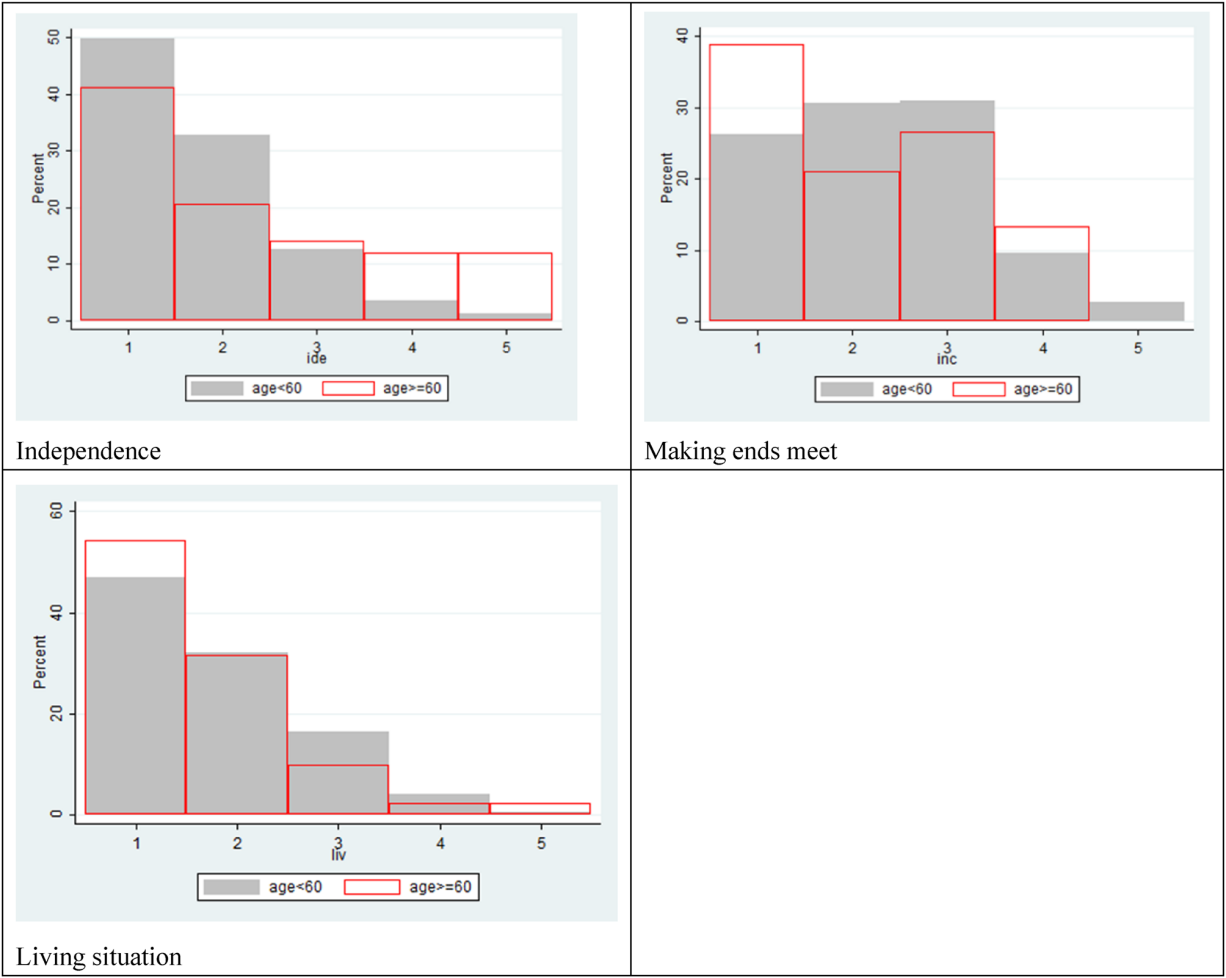
Item distributions between two age groups.

The younger group (n = 230) and older group (n = 92) had 12 and 14 respondents with missing responses for any WOOP item, but the percentages were 5.22% and 15.2% respectively, which was statistically different. Figure 3 shows response distributions between two age groups for each WOOP item. Similar distributions can be seen in ‘mental problem’ and ‘receive support’. The Chi^2^ test suggested younger respondents reported different results for ‘physical problem’, ‘mental problem’, ‘acceptance and resilience’, ‘feeling useful’, ‘independence’.

## Discussion

This study collected WOOP and EQ-5D-5L data from a sample of patients, carers and healthy general public in China. The overall acceptability of WOOP was lower than EQ-5D-5L. The overall response distributions of WOOP items were good except that three items of WOOP did not have any level 5 responses. In comparison, all five response levels were used in EQ-5D-5L, but with the majority of the responses being level 1, suggesting ceiling effects^9^. The items of WOOP did not show strong correlations with EQ-5D-5L items, implying these two instruments measure different constructs. This was confirmed by the EFA analysis that only ‘physical health’ and ‘mental health’ loaded on the same factor with EQ-5D-5L dimensions. Both instruments showed good known-group validity and convergent validity, with EQ-5D-5L having large effect sizes but WOOP had a higher correlation with EQ-VAS.

WOOP items did not show strong correlations with EQ-5D-5L items, suggesting discriminant validity. Similarly, the EFA results indicated that WOOP had only one shared factor with EQ-5D-5L. This finding does not fully agree with the finding reported in the study of Hackert et al. In that study, it was reported that WOOP and EQ-5D-5L tapped into three factors, with two shared factors which were physical health/functioning and mental health/functioning; and a unique factor with only WOOP items^22^. Our results were different in terms of, first, we had a combined factor covering both physical health/functioning and mental health/functioning, which can be interpreted as overall health; second, WOOP had another two unique factors, with the first one covering ‘social contacts’ and ‘receive support’, which can be interpreted as social health/functioning, and the second one including ‘independence’ and ‘making ends meet’ which can be interpreted as autonomy/independence (including financial independence). The difference in the EFA results may be explained by the sample difference, that is, our sample consisted of respondents with different ages and covered patients, carers and healthy general public, which can be seen as a more heterogeneous sample. It is likely that respondents of different ages and respondents with or without a health condition interpreted WOOP items differently. In addition, cultural differences between China and the Netherlands may be another possible reason for this difference. Previous studies have reported that China may have a different conceptual framework compared to Western countries^21^. Despite the different EFA results between this study and the study of Hackert et al., both studies confirmed that WOOP measures a broader construct beyond health. However, it should be noted that 3 items of WOOP (‘acceptance and resilience’, ‘feeling useful’ and ‘living situation’) were not loaded on any factor. Given well-being may be a less concrete concept in China, future study with a large sample size is needed to further validate the measurement structure of well-being measured by WOOP.

Overall, only 10% of all respondents had at least one missing item in our study, which is lower than the percentage reported by Liu et al. Liu et al. reported that approximately 30% of the older people living in rural China had problems with at least one WOOP item as the well-being concepts are more abstract than health related concepts like mobility, pain etc. In both studies, ‘acceptance and resilience’ was found to be an item with a high missing response rate. Liu et al. concluded that these items with missing responses measure more vaguely-defined constructs than items without any missing responses such as ‘physical health’ and ‘living situation’. A qualitative study may be helpful to confirm this. The reduction of acceptability rate can be explained by the sample difference, as Liu et al. interviewed older people in the rural area and our sample was mainly collected in an urban area with respondents younger than 60 years.

Though developed for older people, we found that WOOP can also be used by the younger population. Compared with older people, younger people had fewer missing responses. In addition, compared to the responses of older people, there was a statistical difference between the responses given by the younger people. Specifically, we found that younger people reported lower scores for ‘social contact’, ‘making ends meet’ and ‘living situation’, which suggests these items are relevant for the younger population. Nevertheless, this conclusion should be interpreted with caution, and a content validity study is needed to understand whether these nine items are comprehensive for measuring the well-being of Chinese population.

The known-group validity results suggest the different use of these two instruments. EQ-5D-5L showed a higher effect size when the patient group was compared, but less effect size when comparing carers with general public. This may be because EQ-5D-5L measures HRQoL, which was directly affected by one’s disease status. As a result, patients had a lower utility value when measured by EQ-5D-5L, and the utility values of general public and carers were the same. In comparison, well-being which aims to measure a broader construct, may not be only determined by the disease status, but may be related to other internal and external factors like one’s socioeconomic status and social support etc. When measured by WOOP, we still observed patients reporting lower utility values, but not as low as the utility value measured by EQ-5D-5L. Moreover, we can observe a slight utility difference between the general public and carers when measured by WOOP. This supports the use of WOOP as an instrument that measures a broader scope of QoL, which can be used for quantifying QoL outcomes across healthcare, public health and social care sectors. Since WOOP itself covered both physical health and mental health, Hackert et al. suggested it may be used alone, instead of together with another HRQoL instrument^22^. However, as showed in our study, EQ-5D-5L is a more sensitive instrument when used for measuring patients’ health, that is, the utility difference between patients and healthy general public was larger when measured by EQ-5D-5L. Therefore, we would expect a larger QALY gain if EQ-5D-5L is used. Future studies should explore whether this is the case in a cost-effectiveness study.

This study has some limitations. First, we used the Dutch value set to calculate the WOOP utility. The Dutch value set was based on the preferences of older people who were aged over 65. This contradicts the EQ-5D-5L utility which was established from a taxpayer’s perspective in China. To accurately reflect the preferences of Chinese population, a value set of WOOP based on the preference of Chinese population is necessary. Second, we did not collect the health condition information for general public and carers and assumed they were healthy. In addition, we did not collect much important sociodemographic information, for example, marriage, income, working status, health insurance type etc., which all could affect one’s well-being. We did not include a sufficient sample size for each subgroup of patients, carers, and the general public. Our intention was to recruit a diverse sample to compare the performance of these two instruments in a general setting. However, further research with a larger sample size is required to fully comprehend the psychometric performance of WOOP in these groups. This research should particularly focus on testing the reliability and responsiveness of WOOP in a longitudinal study.

Recently, we have seen a trend to expand the measuring scope of preference based measures, for example, similar to WOOP, EQ-HWB was developed as an instrument to cover both health and well-being and aimed to provide utility value than can be used for economic evaluation across sectors, but the EQ-HWB was not developed specifically for older people^32^. Theoretically, EQ-HWB is a better comparator for WOOP as both instruments simultaneously measure well-being and health. Yet, there are distinct differences between these two instruments, for example, WOOP has some unique items like ‘making ends meet’ and ‘living situation’ which were not included in the EQ-HWB covering 7 domains with 25 items. In a way, WOOP condensed the dimensions of health into the first two items and made room to cover a broader range of well-being concept, e.g., by providing an illustration of each item, the mental health item in WOOP covers constructs related to cognition, negative mental health and mental functioning. There are other key differences between these two instruments, for example, WOOP used a recall period of today, but EQ-HWB used the past seven days. While these developments of new instruments provide alternative instrument choice in research, it is important to examine their differences before applying them in decision-making process.

## Conclusions

WOOP measures a broader construct beyond health and can be used across healthcare, public health and social care sectors in China while EQ-5D-5L is a more sensitive instrument when health is considered alone. Moreover, this study explored and confirmed the potential of using WOOP in a wider population. Future study is needed to explore the content validity of WOOP in China and to compare these two instruments in economic evaluation studies.

## Data Availability

The datasets used and/or analysed during the current study available from the corresponding author on reasonable request.
